# A comparative analysis of fear of cancer recurrence in patients with small renal masses: Active surveillance versus cryoablation

**DOI:** 10.2340/1651-226X.2024.40418

**Published:** 2024-07-22

**Authors:** Rasmine Bak, Theresa Junker, Jørgen B. Jensen, Tau Pelant, Rikke N. Haase, Robert Zachariae, Tommy K. Nielsen

**Affiliations:** a Department of Urology, Aarhus University Hospital, Aarhus, Denmark; b Research and Innovation Unit of Radiology - UNIFY, SDU, Odense, Denmark; c Radiological Research and Innovation Unit, Aarhus University Hospital, Aarhus, Denmark; d Department of Urology, Odense University Hospital, Odense, Denmark; e Department of Clinical Medicine, Aarhus University, Aarhus, Denmark; f Department of Urology, Regional Hospital Gødstrup, Gødstrup, Denmark; g Department of Urology, Aalborg University Hospital, Aalborg, Denmark; h Department of Oncology, Aarhus University Hospital, Aarhus, Denmark; i Department of Psychology and Behavioural Sciences, Aarhus University, Aarhus, Denmark

**Keywords:** Active surveillance, cryoablation, small renal masses, fear of cancer recurrence, kidney cancer

## Abstract

**Background and Purpose:**

The aim of this study was to evaluate and compare the fear of cancer recurrence (FCR) in patients diagnosed with a small renal mass (SRM) and managed with either active surveillance (AS) or minimal invasive renal cryoablation (CA).

**Patients/Material and Methods:**

A total of 398 patients with SRMs (263 AS and 135 CA patients) were retrospectively identified across three institutions and invited to complete the Fear of Cancer Recurrence-Short Form (FCRI-SF) questionnaire.

**Results:**

No statistically significant differences in FCRI-SF score were observed between the AS (mean = 10.9, standard deviation [SD] = 6.9) and CA (mean = 10.2, SD = 7.2) (*p* = 0.559) patients, with the mean scores of both groups being below the suggested clinically significant cut-off of 16. A total of 25% of AS and 28% of CA patients reported sub-clinical or clinical levels of FCR (FCRI-SF score > 16). Within the AS group, a weak negative association between FCR severity and age was observed (*r* = −0.23, *p* = 0.006), and a statistically significant difference in FCRI-SF score between patients aged more or less than 73 years (*p* = 0.009).

**Interpretation:**

FCR levels were comparable between AS and CA patients, suggesting that treatment decisions should prioritise clinical factors. Up to 28% of AS and CA patients report clinically significant FCR, highlighting the importance of considering the possibility of FCR, especially in younger patients.

## Introduction

Over the past three decades, the increased utilisation of computed tomography (CT) has resulted in a rise in the diagnosis of renal cell carcinoma (RCC) and a shift towards earlier-stage tumours [1]. Small renal masses (SRMs) are defined as tumours less than 4 cm in size suspected to be T1a RCC [2]. In recent years, active surveillance (AS) and minimally invasive ablative therapies such as cryoablation (CA) and radiofrequency ablation have emerged as treatment options for SRMs [3]. AS involves the initial monitoring of SRMs using serial abdominal imaging, and delayed intervention is triggered in case of progression during follow-up. AS is generally considered suitable for elderly and comorbid patients with an increased risks associated with surgical management and patients with a strong personal preference [4].

Psychological distress is a significant negative prognostic factor in patients with RCC, associated with poorer survival and quality of life (QoL) [5]. Despite AS being a safe initial treatment for selected patients with SRMs, approximately 40% of these patients eventually require delayed intervention. Of those receiving a delayed intervention, a significant proportion is triggered by the patient’s fear of cancer recurrence (FCR) [6, 7].

FCR is defined as ‘fear, worry, or concern relating to the possibility that cancer will come back or progress’ [8]. While low levels are normal and rational responses to a cancer diagnosis and can be helpful by promoting treatment compliance and healthy lifestyle adaptations, at clinical levels, FCR is associated with impaired QoL, effects on daily functioning, maladaptive healthcare utilisation behaviours, and considerable psychological distress [9]. Several factors have been found to predict higher FCR levels, with younger age being the most consistent predictor [10]. In addition, low general optimism and individuals prone to worry tend to experience higher levels of FCR after cancer treatment [10]. FCR is often triggered or worsened by certain situations, for example, the occurrence of physical symptoms [11–13], an increased focus on cancer and mortality in the media or the social environment, and upcoming medical visits [12]. FCR appears relatively stable over time and can become a lifelong concern if left unaddressed [9, 10].

Only a few studies have investigated the psychological aspects of an AS programme [14–17], and none have examined FCR. Our aim was, therefore, to evaluate the level of FCR in AS patients and compare it to the level of FCR in patients treated with CA.

## Materials and methods

### Study design and population

This retrospective cross-sectional multicentre study identified patients in an AS programme at Aarhus University Hospital (AUH), Denmark, from 2012 to 2023 and at Regional Hospital Gødstrup (RHG) and Aalborg University Hospital (AAUH), Denmark, from 2018 to 2023. Patients were identified through a combination of the Electronic Medical Records and the administrative tool Business Intelligence System by using a search string with referral, diagnosis, and treatment codes. Inclusion criteria encompassed patients with a diagnostic biopsy and patients with suspected cancerous tumours identified by imaging. The study enrolled all patients who had at least one follow-up during the specified timeframe. AS patients diagnosed with hereditary syndromes, renal angiomyolipoma (AML), oncocytoma and Bosniak 2F or lower grade cysts were excluded from inclusion.

CA patients were identified by a retrospective review of the surgery programme between 2021 and 2022 at AUH (AUH is a referral centre and carries out CA for all three institutions). Patients who formerly underwent CA or surgical treatment of the same kidney, former AS programme for the same tumour, oncocytoma, AML, non-residents in Denmark, hereditary syndromes, missing information, metastatic disease, or bilateral diseases were excluded from inclusion. The included AS and CA patients were invited to complete the Fear of Cancer Recurrence Inventory-Short Form (FCRI-SF). Ethical approval was obtained from relevant institutions.

### Fear of cancer recurrence inventory-short form

To enable comparisons with other patient groups, we chose the widely used FCRI-SF. The FCRI-SF is a 9-item subscale with a validated cut-off value for distinguishing ‘normal’ from ‘clinical or pathological’ FCR [18]. It is based on the 42-item FCRI scale [19], which is recognised as one of the strongest psychometrically measures of FCR [20] and has been validated in several languages [21, 22]. We used the Danish version of the FCRI-SF [22] to assess FCR in AS and CA patients. Each item of the FCRI-SF is rated on a 5-point Likert scale ranging from zero (‘Not at all’) to four (‘A great deal’), with possible total scores ranging from 0 to 36. One item (no. 5) is reversed before summing the score. A cut-off value for clinical FCR is given in the FCRI-SF, defined by a score of 16 or higher. Additionally, scores of 13 and 22 have also been suggested as clinical cut-offs [18]. One item (no. 5) was modified from *‘I believe that I am cured, and that the cancer will not come back’ to ‘I have confidence in my surveillance program, and I do not think that my small renal mass will progress’* for the AS patients. The modification aims to enhance the comprehensive understanding of FCR as the item may not be meaningful to AS patients with active cancer who do not experience themselves as cured.

### Clinical data

The follow-up time was defined as the months from AS decision or CA treatment to the date of survey response. Comorbidities were assessed using the age-adjusted Charlson Comorbidity Index Score (ACCI) [23] at the time of diagnosis. The overall GR for the AS patients was calculated as the size difference between the first and last scan divided by the time interval between the two scans. The GR was expressed as the rate of change in millimetres per year. Furthermore, information on body mass index (BMI), initial creatinine level, non-kidney cancer status, initial biopsy status and tumour details was collected.

### Statistics

Categorical variables were compared using the Pearson chi-square test, while continuous variables were analysed using t-tests for independent samples. The data analyses were performed under the assumption of normally distributed differences. In case of non-normal distribution of the differences, a Wilcoxon-rank sum test was performed. A *p*-value < 0.05 was considered statistically significant.

Missing values on the FCRI-SF were handled similarly to previous validation studies [19, 21]. A total FCRI-SF score was excluded if more than 50% of the items were missing or if it had adopted a ‘0’ response pattern for all of the FCRI-SF items, including the item that was scored in the reverse (item no. 5). For the remaining scores, missing data were imputed with the mean score of the remaining items completed by the participant as the Cronbach’s alpha > 0.7. When assessing differences in FCRI-SF scores between two groups, the groups were categorised using the median value as the threshold. The mean value was used when comparing growth rate, as outliers resulted in an uneven distribution. When comparing ages, they were stratified into three groups to provide more informative output. All analyses were conducted using Rstudio 2022.07.2 + 576.

### Results

A total of 263 AS patients were invited to participate in the study. Of these, 144 patients (56%) responded. One participant was excluded due to a ‘0’ response pattern. None were excluded due to > 50% missing values. Two patients had one missing answer imputed with the mean of the remaining items. Thus, a total of 143 patients, corresponding to a response rate of 54%, were included in the analysis. There were no significant differences between responders and non-responders regarding gender (male-to-female ratio: 1.7:1 vs. 1.8:1; *p* = 0.771). Responders were significantly younger than non-responders (71 years vs. 73 years; *p* = 0.036).

A total of 254 CA patients were identified of which 119 patients were excluded due to re-cryoablation or former treatment in the same kidney (*n* = 28), cross-over from prior AS for the same tumour (*n* = 27), oncocytoma or AML (*n* = 36), bilateral disease (*n* = 14), not resident of Denmark (*n* = 7), hereditary disease (*n* = 3), metastatic disease (*n* = 3), or missing information (*n* = 1), resulting in a total of 135 patients, who were invited to participate in the study. A total of 48 patients did not respond, and two patients were excluded due to a ‘0’ response pattern. Thus, a total of 85 patients were included in the analysis, corresponding to a response rate of 63%. The difference between responders and non-responders regarding gender did not reach statistical significance (male-to-female ratio: 2.7:1 vs. 4.3:1; *p* = 0.387). Responders were significantly older than non-responders (65 years vs. 60 years; *p* = 0.008).

### Demographics

AS patients were significantly older (71 years vs. 66 years; *p* < 0.001) at diagnosis, had significantly more comorbidities (ACCI 5.0 vs. 3.7; *p* < 0.001) and a significantly smaller initial tumour size (15 mm vs. 23 mm; *p* < 0.001), compared with patients treated with CA. The differences between the two groups regarding gender (*p* = 0.160), BMI (*p* = 0.734), initial creatinine levels (*p* = 0.139), non-kidney cancer status (*p* = 0.648), and tumour location (*p* = 0.066) did not reach statistical significance. The proportion of patients who initially underwent biopsy was significantly lower in the AS group compared to the CA group (29% vs. 100%; *p* < 0.001). Additionally, AS patients presented with a higher rate of non-diagnostic biopsies in terms of histology (54% vs. 0%) when compared to CA patients. AS patients had a significantly longer follow-up period compared to the CA patients (23 months vs. 14 months; *p* < 0.001). Demographic and clinical information are presented in Table 1.

**Table 1:** Patient and tumour characteristics for AS and CA patients.

	AS (*n* = 143)	CA (*n* = 85)	*P*
**Mean age, year (SD)**	71 (9.2)	66 (10.42)	< 0.001
**Male, *n* (%)**	90 (62.9)	62 (72.9)	0.160
**Mean BMI, kg/m**^**2**^ **(SD)**	28 (5.8)	29 (5.5)	0.734
**Mean ACCI score (SD)**	5.0 (2.5)	3.7 (2.2)	< 0.001
**Mean initial creatinine, µmol/l (SD)**	92 (59.8)	82 (20.4)	0.139
**Non-kidney cancer status, *n* (%)**			0.648
Never had cancer	78 (54.5)	49 (57.6)	
Current or former cancer	56 (39.2)	33 (38.8)	
Current or former metastasis	9 (6.3)	3 (3.5)	
**Location, *n* (%)**			0.066
Right	70 (49.0)	39 (45.9)	
Left	62 (43.4)	46 (54.1)	
Bilateral/Multiple	11 (7.7)	0 (0.0)	
**Initial tumour size, mm (SD)**	15 (10.4)	23 (8.3)	< 0.001
**Initial biopsy, *n* (%)**	41 (28.7)	85 (100)	< 0.001
**Histology, *n* (%)**			< 0.001
Clear cell	4 (9.8)	43 (50.6)	
Chromophobe	3 (7.3)	4 (4.7)	
Papillary	7 (17.1)	28 (32.9)	
Other histology	5 (12.2)	10 (11.8)	
Non-diagnostic biopsy	22 (53.7)	0 (0.0)	
**Mean follow-up time, months (SD)**	23 (18.5)	14 (6.4)	< 0.001

SD: standard deviation; BMI: body mass index; ACCI: age-adjusted charlson comorbidity index score.

### Fear of cancer recurrence for active surveillance and cryoablation patients

No statistically significant difference was found in mean FCRI-SF score between the AS and CA group (10.9 vs. 10.2; *p* = 0.559). The distribution of patients based on various cut-off scores was similar in the two groups (χ^2^ = 0.41, *p* = 0 938). A total of 39% of AS and 40% of CA patients presented with FCR as defined by a cut-off of ≥ 13. With a cut-off of ≥ 16, a total of 25% of AS and 28% of CA patients present with clinical FCR. A total of 7% of AS and 8% of CA patients presented with clinically severe FCR with a cut-off of ≥ 22 (Table 2).

**Table 2:** FCRI-SF scores for AS and CA patients.

	AS (*n* = 143)	CA (*n* = 85)	*P*
**Mean FCRI-SF score, *n* (%)**	10.9 (6.9)	10.2 (7.2)	0.559
**FCRI-SF cut-off’s, *n* (%)**			0.938
< 13	87 (60.8)	51 (60.0)	
≥ 13 – < 16	20 (14.0)	10 (11.8)	
≥ 16 – < 22	26 (18.2)	17 (20.0)	
≥ 22	10 (7.0)	7 (8.2)	

FCRI-SF: fear of cancer recurrence inventory-short form; AS: active surveillance; CA: cryoablation.

**Figure 1:**
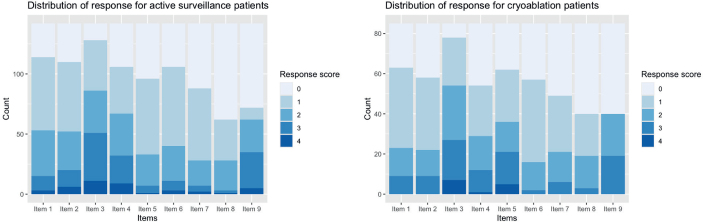
The distribution of response (score between 0 and 4) for each item in the FCRI-SF for both AS and CA group. FCRI-SF: fear of cancer recurrence inventory-short form.

The response patterns for the two groups were similar, with an equal distribution of responses ranging from zero to four (Figure 1). Item three, which assesses patients’ beliefs regarding the normality of being worried or anxious about the possibility of the cancer was the item most endorsed in both groups, while items eight and nine, which are concerned with the time spent contemplating cancer recurrence and its duration, were the least endorsed items.

### Fear of cancer recurrence for active surveillance patients

**Figure 2:**
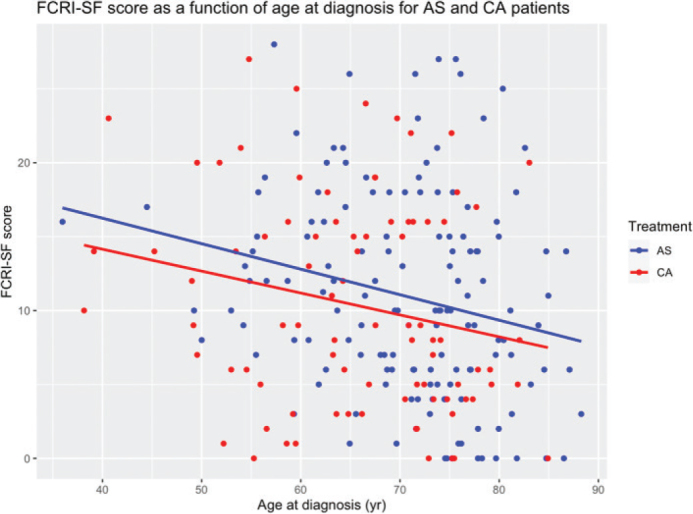
Relationship between FCRI-SF score and age at diagnoses for both the AS and CA group. FCRI-SF: fear of cancer recurrence inventory-short form; AS: active surveillance; CA: cryoablation.

When comparing subgroups of AS, patients younger than 60 years presented significantly higher FCRI-SF scores compared to those aged 60–70 years and those over 70 years (13.3 vs. 11.9 vs. 10.0, respectively; *p* = 0.03). In addition, older patients in both the AS and CA group demonstrated a tendency towards lower FCRI-SF scores, which was consistent with the small negative association found between FCRI-SF score and age (*r* = −0.23, *p* = 0.006) (Figure 2). No statistically significant associations were observed between FCRI-SF scores and gender (*p* = 0.896), initial biopsy status (*p* = 0.147), comorbidities (*p* = 0.598, *r* = 0.064), time to follow-up (*p* = 0.127, *r* = −0.14), initial tumour size (*p* = 0.512, *r* = −0.025), growth rate (*p* = 0.754, *r* = 0.011), or former cancer status (*p* = 0.373) (Table 3).

**Table 3:** FCRI-SF scores within AS subgroups.

	SUBGROUP			*P*	*r*
**Age at diagnosis**	**< 60 years**	**60–70 years**	**≥ 70 years**		
No. of patients	19	39	85		
FCRI-SF score (SD)	13.3 (5.9)	11.9 (6.1)	10.0 (7.4)	0.03	-
**Gender**	**Female**	**Male**			
No. of patients	53	90			
FCRI-SF score (SD)	11.0 (7.5)	10.9 (6.6)		0.896	-
**Initial biopsy**	**Yes**	**No**			
No. of patients	41	102			
FCRI-SF score (SD)	12.3 (7.1)	10.4 (6.9)		0.147	-
**ACCI score**	**Score ≤ 5**	**Score > 5**			
No. of patients	64	79			
FCRI-SF score (SD)	10.6 (6.2)	11.2 (7.5)		0.598	0.064
**Follow-up time**	**≤ 18 months**	**> 18 months**			
No. of patients	72	71			
FCRI-SF score (SD)	11.8 (7.1)	10.1 (6.8)		0.127	-0.14
**Tumour size at diagnosis**	**≤ 12 mm**	**> 12 mm**			
No. of patients	63	80			
FCRI-SF score (SD)	10.5 (6.7)	11.3 (7.2)		0.512	-0.025
**Growth rate**	**≤ 0.7 mm/year**	**> 0.7 mm/year**			
No. patients	87	54			
FCRI-SF score (SD)	11.1 (7.2)	10.7 (6.6)		0.754	0.011
**Former cancer diagnosis**	**Never**	**Yes**			
No. patients	78	65			
FCRI-SF score (SD)	10.5 (6.2)	11.5 (7.7)		0.373	-

FCRI-SF: Fear of Cancer Recurrence Inventory-Short Form; SD: standard deviation; ACCI: age-adjusted Charlson Comorbidity Index Score; AS: active surveillance.

## Discussion

No significant difference in FCR was observed between AS and CA patients, with approximately 25–28% of all patients experiencing clinical FCR with a cut-off of ≥ 16. The mean FCRI-SF scores were 10.9 for AS and 10.2 for CA patients, aligning with the lowest mean score of 10.4 for endometrial cancer presented in a meta-analysis of Smith et al. (mean FCRI-SF = 15.8) [24] and the lowest mean score of 11.2 for prostate cancer in a meta-analysis of Luigjes-Huizer et al. (mean FCRI-SF = 14.8) [25]. Scores of 13, 16 and 22 have been suggested as cut-offs for clinical FCR [18]. Scores of ≥ 13 suggest the possibility of clinical FCR, scores ≥ 16 indicate the likelihood of clinical FCR and scores ≥ 22 indicate a clinically severe FCR requiring specialised intervention [25]. Our results showed lower proportions above these cut-offs, as 39–40% scored ≥ 13, 25–28% scored ≥ 16, and 7–8% scored ≥ 22, when compared to Smith et al. (54, 43 and 30%) [24] and Luigjes-Huizer et al. (59, 45 and 19%) [25]. In addition, we observed a weak negative correlation between age and FCR severity in AS patients (*r* = −0.23, *p* = 0.006), and a statistically significant difference in FCRI-SF scores between AS patients aged under 60 years, and those aged 60–70 years, and those aged over 70 years (*p* = 0.03). Our correlation aligns with similar findings in other non-kidney cancer studies with *r* values ranging from −0.29 to −0.31 [19, 21, 22]. Similarly, Luigjes-Huizer et al. [25] and Smith et al. [24] found a significant association between FCR severity and younger age as well as female gender. Although AS and CA patients seem less affected by FCR compared to other cancer types, up to 28% experience sub-clinical or clinical levels of FCR, underscoring its clinical importance, especially in younger patients. These findings highlight the importance of healthcare providers addressing FCR through targeted discussions and interventions in routine patient care. Proactive conversations of coping strategies and support can contribute to holistic care of patients and enhancing the patient–doctor relationship. By acknowledging and addressing FCR, healthcare providers contribute to the overall QoL for individuals navigating the challenges of living with cancer.

Clinical FCR correlates with reduced overall QoL, psychological distress, impaired physical, emotional, cognitive, and social functioning, as well as increased healthcare costs [10, 18, 26–29]. In RCC patients, psychological distress is a significant prognostic factor associated with poorer survival rates (Positive affect: HR = 0.90; *p* = 0.009, Depressive symptom: HR = 1.03; *p* = 0.013) [5], highlighting the importance of recognising psychological distress and reduced QoL in affected patients. Notably, Bergerot et al. [30] found significant associations between higher levels of distress and both female gender and younger age, which aligns well with our findings of an association between elevated FCR scores and younger age. This suggest potential advantages in implementing a more thorough follow-up programme for patients with higher FCR levels, particularly among younger patients, focusing on information and coping, as Parker et al. found an association between higher levels of illness uncertainty and reduced QoL in AS patients [14]. Comparable with our observation of a similar FCRI-SF score between AS and CA patients, two studies reported similar levels of psychological distress and QoL in AS patients and those initially treated with surgery or ablation [15, 16]. These findings suggest that treatment decisions should prioritise clinical aspects, potentially giving importance to AS when the two modalities are equally viable options, as AS does not impair renal function.

To our knowledge this study is the first study to investigate and compare FCR in AS and CA patients, providing valuable insights in relation to treatment decision concerning patients diagnosed with SRMs. In addition, this study addresses a common underrepresentation of elderly in FCR research, as only 23% of patients included in the meta-analysis of Luigjes-Huizer et al. were ≥ 70 years old [25]. This study contributes with significant information in this domain, as 58% of AS and 42% of CA patients falling within the ≥70 age group. Some limitations should also be acknowledged. Firstly, the suitability of FCRI for evaluating fear of cancer progression (FoP) as well as FCR remains unclear [24]. Some FCRI items may inadequately address FoP in the present cancer population. For instance, the statement in item 5, *‘I believe that I am cured, and that the cancer will not come back’* may not be meaningful to cancer patients with active cancer who do not experience themselves as cured, even if they consider the likelihood of cancer progression to be low. To address this issue, we modified the questionnaire, changing the wording to *‘I have confidence in my surveillance program, and I do not think that my small renal mass will progress’*. While this adaptation may complicate direct comparisons with standard FCRI-SF studies and the CA patients, it stands as a strength in our context, ensuring that the modified questionnaire resonates with the unique experiences and concerns of the AS patients, and thereby providing more relevant insights into their FCR. Another concern related to the use of FCRI-SF as a measure of FCR is the lack of some key characteristics of clinical FCR [31]. Further research with, for example, FCRI may be needed. Secondly, the potential influence of age on FCR severity is noteworthy, considering the significant age difference between the AS and CA patients (*p* < 0.001) in our study. This age difference may have led to an underestimation of FCRI-SF scores for AS patients compared to CA patients, given the negative association between age and FCRI-SF scores. Within the AS group, non-responders were significantly older than responders (*p* = 0.036), potentially overestimating FCR in the AS group. Conversely, non-responders were significantly younger than responders within the CA group (*p* = 0.008), potentially resulting in an underestimation of FCR. Thus, age-related differences within and between the groups may have introduced biases, which can impact the interpretation of FCRI-SF scores. The non-kidney cancer status was consistent between the two groups and no correlations were found between FCR and other tested variables, including gender (*p* = 0.896) and time since diagnosis (more or less than 18 months, *p* = 0.127). This finding is supported by Luigjes-Huizer et al., who also found no significant association between FCR severity and time since diagnosis [25], suggesting that FCR persists over time without intervention or treatment. This enables the possibility of comparing patients from different inclusion periods (2012–2023 for AS patients vs. 2021–2022 for CA patients). Furthermore, differences in initial biopsy status and non-diagnostic rates between AS and CA patients may have influenced the FCR scores due to differences in tumour histology knowledge. Biopsying AS patients poses significant challenges, primarily due to the high inconclusive rate. Hence, it is common clinical practice to include patients in AS based on suspected cancerous tumours, and inclusion does not require an initial biopsy. The low biopsy rate among AS patients presents a challenge, as a confirmed diagnosis via biopsy would be optimal for both the patient and the practitioner [32]. Thirdly, the retrospective study design lacked a timeline, limiting our ability to investigate changes in FCR over time. In addition, the study’s retrospective design may have introduced confounding results by indication and selection bias as the patients were not randomised into their treatment modality. Finally, our study missed information regarding family history of cancer, psychiatric history, and current psychological status, which could potentially have influenced our results.

## Conclusion

FCR levels were comparable between AS and CA patients, suggesting that treatment decisions should prioritise clinical factors. Up to 28% of AS and CA patients experience clinical FCR, highlighting the importance of considering the possibility of FCR, especially in younger patients. Further research, including prospective longitudinal studies, is needed to confirm these findings and explore the underlying mechanisms and interventional effects.

## Data Availability

The data utilised in this article has been collected using REDCap and can be obtained from there. Please contact Corresponding author Rasmine Bak for access to the data.
